# Machine learning-based prediction of cerebral artery territorial infarcts in acute ischemic stroke using non-contrast CT

**DOI:** 10.3389/fneur.2026.1794563

**Published:** 2026-07-24

**Authors:** Yang Guo, Shui Liu, Hongmei Sun, Xiukun Jin, Yan Zhang, Jian Sun, Zhiqun Wang, Lvming Zhang

**Affiliations:** 1 Department of Radiology, Peking University Aerospace Clinical Medical College, Beijing, China; 2 Department of Radiology, Aerospace Center Hospital, Beijing, China; 3 Department of Neurology, Aerospace Center Hospital, Beijing, China

**Keywords:** infarct localization, label annotation, leak-proof pipeline, machine learning, non-contrast CT, prediction model

## Abstract

**Background:**

Ischemic stroke is a leading global cause of death and disability. Accurate, rapid infarct territory localization is critical for timely treatment, but ultra-early ischemic changes on non-contrast CT (NCCT) are subtle and highly physician-dependent.

**Methods:**

This retrospective study enrolled acute ischemic stroke patients with single-territory infarction. Clinical data and NCCT images were collected. Quantitative imaging features (HU values, infarct volume, spatial distribution) were extracted from vascular perfusion territories. A strict leak-proof pipeline was adopted: dataset stratification, missing value imputation, scaling, and Synthetic Minority Oversampling Technique (SMOTE) were applied only to the training set, full feature retention (VIF < 5), and Logistic Regression (LR) and XGBoost models were constructed. Standardized labeling was performed. Anterior cerebral artery (ACA), middle cerebral artery (MCA), and posterior cerebral artery (PCA) territories were defined by two neuroradiologists using diffusion-weighted imaging (DWI) (gold standard, Kappa analysis). Multi-territory infarcts were excluded. TotalSegmentator segmented parenchymal perfusion territories. DWI infarct ROIs were registered to NCCT. Model performance was assessed by Area under the receiver operating characteristic curve (AUC), sensitivity, specificity, precision and calibration (Brier score, slope), with DeLong-Bonferroni tests. Robust validation used 5-fold repeated stratified cross-validation (10 repetitions).

**Results:**

A total of 118 eligible patients were enrolled (median age: 72.0 years; IQR: 65.0–79.0; 85.6% male). All patients received NCCT within 24 h of stroke onset, with an average NIHSS score of 6.05 ± 3.26. Inter-rater Kappa for infarct labeling reached 0.89 (excellent agreement). On the test set, XGBoost yielded AUCs of 0.92 (ACA), 0.87 (MCA), and 0.95 (PCA), with a cross-validated mean AUC of 0.94 ± 0.03. Mild overfitting was noted due to a performance drop from training to test data. LR produced AUCs ranging 0.78–0.86 with balanced sensitivity. For left/right/bilateral infarct classification, XGBoost achieved a higher mean AUC (0.90 vs. 0.82, *p* < 0.01). Both models exhibited good calibration.

**Conclusion:**

XGBoost demonstrates excellent diagnostic performance for infarct territory localization, whereas LR offers stable sensitivity. Strict protocols and robust validation support its clinical auxiliary value. Future studies will incorporate multi-center external validation and multi-territory infarcts to improve generalizability.

## 1 Introduction

Ischemic stroke ranks as the second leading cause of death and the third leading cause of disability worldwide, claiming nearly 7 million deaths annually (1–4). In recent years, driven by population growth and aging trends, the global incidence and prevalence of stroke have been steadily increasing globally (5–7). Currently, intravenous thrombolysis and endovascular thrombectomy represent the most effective reperfusion therapies during the acute phase (8–10), but they require treatment within a narrow time window (usually within 4.5–6 h after symptom onset) (11, 12). Therefore, rapid and accurate imaging assessment such as CT, MRI and etiological classification are crucial for guiding clinical decision-making.

Non-contrast CT (NCCT) has become the imaging modality of choice for patients with suspected stroke due to its rapid acquisition and cost-effectiveness (13, 14). Although NCCT can effectively rule out cerebral hemorrhage and detect early ischemic signs such as manifestations related to the ASPECTS score (15, 16), ultra-early ischemic changes are often subtle and highly dependent on physician experience. Determining infarction within specific cerebral arterial perfusion territories [anterior cerebral artery (ACA), middle cerebral artery (MCA), and posterior cerebral artery (PCA)] is particularly challenging (17). As infarcts in different territories correspond to different therapeutic strategies and prognoses, rapid and accurate localization holds significant clinical value.

Machine learning (ML) has demonstrated significant advantages in medical image analysis for assisting diagnosis, risk prediction, and prognostic assessment through data-driven models (18, 19). ML models, such as Random Forest and Support Vector Machines (SVM), have been shown to outperform traditional methods in stroke risk prediction (20, 21) and prognostic assessment (22, 23). They are often more accurate, especially for specific populations such as Chinese patients (24–26). However, NCCT image-based ML models for differentiating infarcts in cerebral arterial territories are still lacking, and available studies mostly focus on multimodal imaging or overall risk prediction (27, 28).

In this study, we propose a “region-specific stratified modeling framework” strategy by segmenting NCCT images of ischemic stroke patients using the TotalSegmentator tool (29, 30) to construct predictive models for infarcts in the ACA, MCA, and PCA vascular territories using logistic regression (LR) and XGBoost algorithms. Validated through dataset splitting into training, validation, and test sets, the aim is to develop tools that assist clinicians in rapidly identifying the involved arteries, thereby saving time for precise treatment and providing a basis for clinical decision-making.

## 2 Materials and methods

### 2.1 Ethics statement

This study was approved by the Ethics Committee of Aerospace Central Hospital (Ethical Review Approval No. 2022(009)). The study was conducted in accordance with the Declaration of Helsinki (2013 revision). Given the retrospective nature of the study, written informed consent was waived by the ethics committee, and all patient data were de-identified during collection and analysis to protect privacy. All clinical and imaging data were stored in a password-protected hospital server with access restricted only to the research team.

### 2.2 Study population

This retrospective study included ischemic stroke patients admitted to Aerospace Central Hospital between February 2022 and December 2024. Inclusion criteria were: (1) confirmed acute ischemic stroke via clinical evaluation and 3.0 T diffusion-weighted imaging (DWI) (gold standard); (2) NCCT completed within 24 h of symptom onset with complete imaging data; (3) complete clinical records (age, gender, medical history, NIHSS score at admission); (4) single-territory infarct (ACA/MCA/PCA only, multi-territory/multifocal infarcts are excluded to ensure mutually exclusive labels). Multifocal infarcts are excluded for the first model to ensure mutually exclusive labels. Multifocal infarction data were utilized for the subsequent separate prediction model of bilateral cerebral infarction. (5) no severe NCCT artefacts or substandard image quality. Exclusion criteria were: (1) interval >24 h between symptom onset and NCCT scan; (2) concurrent brain tumors, intracerebral hemorrhage, or other intracranial space-occupying lesions; (3) missing or untraceable clinical/imaging data; (4) history of prior cerebral infarction or neurological disease affecting imaging interpretation.(5) Multi-territory infarcts were excluded to ensure mutually exclusive single territory labels for ACA/MCA/PCA classification; mixed-territory lesions cannot be assigned to a single vascular supply and would cause ambiguous gold-standard labels. Basilar artery infarcts were excluded because TotalSegmentator’s built-in perfusion atlas lacks standardized basilar territory partitioning, leading to inaccurate automatic segmentation of brain parenchyma. This study was designed as an exploratory research focusing on single-territory stroke initially.

**Figure 1:**
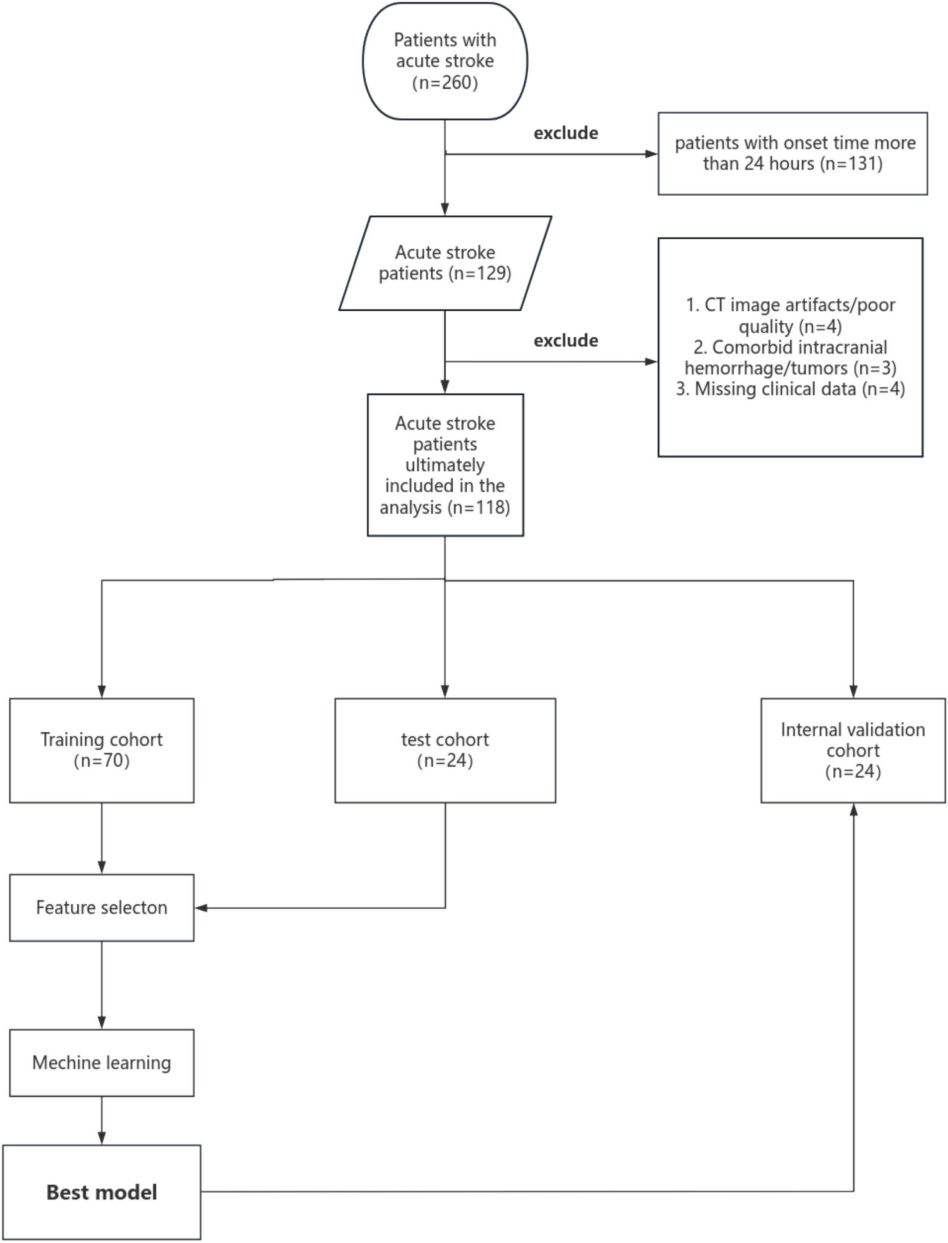
Study flowchart of the study design.

A total of 260 patients were initially screened, 131 were excluded for symptom-to-CT interval >24 h, and 11 were further excluded for NCCI image artifacts/poor quality (*n* = 4), missing data (*n* = 4), and severe comorbid intracranial hemorrhage/tumors (*n* = 3). Ultimately, 118 eligible acute ischemic stroke patients with single-territory infarcts were included, divided into three groups by infarct territory (confirmed via DWI): ACA infarction (*n* = 37), MCA infarction (*n* = 53), PCA infarction (*n* = 28). A 5-fold cross-validation was adopted to randomly partition the training and validation sets, and an independent in-center test dataset was utilized, which was excluded from the model training process. The patient recruitment flowchart is shown in Figure 1.

### 2.3 Label definition and standardized annotation protocol

All labels were defined based on clinical practice and imaging evidence (MRI-DWI as the gold standard) with a standardized annotation protocol conducted by two senior neuroradiologists. A third senior neuroradiologist was consulted to resolve discrepancies, and Cohen’s Kappa coefficient was used to evaluate inter-rater consistency (Kappa >0.8 indicated excellent consistency, 0.6–0.8 good, 0.4–0.6 moderate, <0.4 poor).

#### 2.3.1 Acute vs. non-acute infarction labels

Acute infarction was defined by combined clinical and imaging criteria: Clinical criterion: Symptom onset-to-imaging (NCCT/DWI) interval ≤7 days; Imaging criteria: (a) DWI: hyperintense infarct foci with corresponding hypointensity on apparent diffusion coefficient (ADC) maps; (b) NCCT: focal hypodensity in the cerebral parenchyma (HU value reduction >10 compared to contralateral normal tissue) or sulcal effacement without obvious encephalomalacia. Non-acute infarction was defined as: Symptom onset-to-imaging interval >7 days; DWI: no hyperintense foci, with encephalomalacia (cystic change) on T1WI/T2WI; NCCT: obvious encephalomalacia with focal parenchymal volume loss. The acute/non-acute prediction task was defined as a binary classification task.

#### 2.3.2 Left/right/bilateral infarct labels

Defined by the anatomical location of infarct foci on DWI: left (infarct in the left cerebral hemisphere), right (infarct in the right cerebral hemisphere), bilateral (infarct in both hemispheres). Bilateral infarcts were included as an independent category, and the task was defined as a multi-class classification task (left vs. right vs. bilateral).

#### 2.3.3 ACA/MCA/PCA territorial labels

Territorial labels were assigned by visual adjudication of 3.0 T MRI-DWI images (gold standard) based on the Talairach and Tournoux brain atlas (cerebral arterial perfusion territory division). The annotation steps were: Two neuroradiologists independently reviewed DWI images to identify infarct foci and determine the corresponding perfused cerebral arterial territory (ACA/MCA/PCA) based on anatomical location. Discrepancies were documented and resolved via consensus discussion with the third neuroradiologist. Inter-rater consistency was calculated, with a final Kappa coefficient of 0.89 (95% CI: 0.82–0.96, excellent consistency). ACA perfusion territory: Medial frontal lobe, medial parietal lobe, corpus callosum; MCA perfusion territory: Lateral frontal lobe, parietal lobe, temporal lobe, insula; PCA perfusion territory: Occipital lobe, medial temporal lobe, thalamus. Multi-territory infarcts were excluded to ensure label mutual exclusivity, and the prediction task was defined as a multi-class classification task (ACA vs. MCA vs. PCA) for single-territory infarcts. In this study, the ischemic infarct regions were segmented and classified according to the vascular supply territories of the ACA, MCA, and PCA. Notably, infarct lesions located within the vascular territory of the basilar artery were explicitly excluded from this regional partitioning strategy and were not included in the subsequent regional model construction and analysis.

### 2.4 Data collection and missing value handling

Clinical features were collected from electronic medical records and standardized case report forms: (1) demographic information (age, gender); (2) comorbidities (hypertension, diabetes mellitus, hyperlipidemia, atrial fibrillation, coronary heart disease); (3) neurological function (NIHSS score at admission). Imaging features were extracted from NCCT as described in Section 2.5.

Missing data assessment showed that only three variables had minor missing values (all <5%): hyperlipidemia (2.54%, *n* = 3), atrial fibrillation (1.69%, *n* = 2), coronary heart disease (0.85%, *n* = 1). Median imputation (for continuous variables) and mode imputation (for categorical variables) were used for missing value filling, with the imputation model fitted exclusively on the training set and applied to the validation/test sets (no re-fitting) to prevent data leakage. No other variables had missing data.

### 2.5 Neuroimaging acquisition and feature extraction

#### 2.5.1 NCCT and MRI-DWI acquisition parameters

*NCCT acquisition*: All scans were performed on a Siemens Somatom Force dual-source CT scanner with standardized acquisition and reconstruction parameters (no ambiguity): Acquisition parameters: Tube voltage 120 kVp, tube current 170 mA, collimation 64 × 0.6 mm, pitch 0.984; Reconstruction parameters: Slice thickness 1.25 mm, slice spacing 0.625 mm (contiguous reconstruction), soft tissue kernel (B30f), axial coverage from the skull base to the top of the cerebrum. MRI-DWI acquisition (gold standard): All scans were performed on a 3.0 T Siemens Magnetom Skyra scanner with the following protocol: axial T1WI, T2WI, FLAIR, DWI (b-values = 0 and 1,000 s/mm^2^) with ADC maps, sagittal T1WI. Parameters: (1) Axial T1WI: TR = 450 ms, TE = 11 ms, flip angle = 150°, slice thickness = 5.0 mm, gap = 1.5 mm; (2) Axial T2WI: TR = 4,000 ms, TE = 100 ms, flip angle = 150°, slice thickness = 5.0 mm, gap = 1.5 mm; (3) Axial FLAIR: TR = 8,500 ms, TE = 81 ms, TI = 2,400 ms, flip angle = 150°, slice thickness = 5.0 mm, gap = 1.5 mm; (4) Axial DWI: TR = 4,200 ms, TE = 75 ms, flip angle = 150°, slice thickness = 5.0 mm, gap = 1.5 mm.

#### 2.5.2 TotalSegmentator segmentation

The open-source deep learning tool TotalSegmentator (v1.5.6) was used to segment cerebral parenchymal perfusion territories of the ACA, MCA, and PCA (not vascular lumens/vascular trees)—a critical clarification from standard vascular segmentation. This tool has been fully validated in peer-reviewed studies (28, 29) for accurate segmentation of cerebral anatomical structures and perfusion territories on CT images, with its segmentation logic strictly aligned with the Talairach and Tournoux brain atlas (the gold standard for cerebral arterial perfusion territory division), ensuring inherent anatomical validity for vascular territory segmentation in this study. The segmentation was based on the MNI152 brain template and trained on cerebral arterial perfusion territory atlases, with the segmentation steps: NCCT images were converted to NIfTI format and preprocessed (bias field correction, skull stripping) using Simple ITK (v2.2.1); TotalSegmentator was run with the custom parameter --task cerebralperfusion_territories to segment ACA, MCA, and PCA perfusion territories (cerebral parenchyma); To verify and ensure the effectiveness of segmentation results (which directly underpin subsequent feature extraction and model prediction), strict post-segmentation quality control (QC) protocols were implemented: segmentation results were independently inspected by two senior neuroradiologists with over 10 years of neuroimaging experience, with incorrect segmentations (e.g., mismatched anatomical boundaries, incomplete parenchymal coverage) corrected via 3D Slicer (v5.4.0) and unresolved discrepancies resolved by consensus with a third senior neuroradiologist. This comprehensive QC process guaranteed the high accuracy and consistency of the final perfusion territory segmentation masks, and the excellent reproducibility of subsequent imaging feature extraction (ICC > 0.90, Section 3.1) further validated the reliability of the segmentation results.

#### 2.5.3 DWI-infarct label mapping and NCCT infarct ROI definition

To ensure accurate alignment between the gold-standard infarct labeling (from MRI-DWI) and NCCT-based feature extraction, we adopted a direct label mapping strategy (without rigid registration) by integrating MRI-derived infarct annotations with NCCT perfusion territory segmentation. The detailed process and quality control (QC) steps are as follows: MRI-DWI Infarct Label Annotation: Two senior neuroradiologists independently identified infarct foci on 3.0 T MRI-DWI images (b = 1,000 s/mm^2^) and manually annotated the infarct boundary using 3D Slicer (v5.4.0). The annotation was guided by ADC maps (to confirm acute ischemia) and the Talairach & Tournoux brain atlas (to ensure anatomical consistency). Discrepancies between radiologists were resolved via consensus with a third senior neuroradiologist, and the final infarct labels (binary masks: 1 = infarct, 0 = normal tissue) were saved in NIfTI format. NCCT Perfusion Territory Segmentation: As described in Section 2.5.2, TotalSegmentator (v1.5.6) was used to segment ACA/MCA/PCA cerebral parenchymal perfusion territories from NCCT images. Post-segmentation manual correction was performed to ensure the segmentation masks accurately matched the anatomical boundaries of each vascular territory (no vascular lumen inclusion). Infarct ROI Mapping via Label Merging: The MRI-derived infarct labels were directly mapped to the NCCT perfusion territory masks through voxel-wise logical intersection (implemented in Python with SimpleITK v2.2.1). Specifically: The spatial resolution and anatomical coverage of MRI-DWI infarct labels were standardized to match NCCT images (1.25 mm slice thickness, 0.625 mm interval) to ensure voxel-level correspondence; For each NCCT perfusion territory mask (ACA/MCA/PCA), the overlapping region with the MRI-DWI infarct label was defined as the NCCT infarct ROI (i.e., only the infarct tissue within the corresponding vascular territory was retained); Non-infarct regions, cerebrospinal fluid, and extracerebral tissues were excluded from the ROI to avoid feature contamination.

#### 2.5.4 Standardized imaging feature extraction

Imaging features were extracted from the NCCT infarct ROIs (within ACA/MCA/PCA perfusion territories) using GE AW Server 5.7 (commercial image analysis software), with detailed, reproducible extraction rules (no ambiguity) and manual correction protocols: HU value features: Mean, median, and maximum HU values within the infarct ROI; threshold definition: HU values were measured in the core infarct area (excluding normal parenchyma and cerebrospinal fluid), with manual correction to exclude artefacts such as bone and metal; Infarct volume: Calculated as slice thickness × sum of infarct areas on consecutive axial slices (GE AW Server automatic volume calculation), with manual correction for slice misalignment or partial volume effects; Spatial distribution features: Binary indicators (0/1) for infarct location (left/right) and extent (superficial cortical/deep subcortical/both), defined based on the Talairach and Tournoux atlas. All feature extraction was performed by a single trained radiologist, with 10% of cases randomly selected for re-extraction to test reproducibility (intra-class correlation coefficient [ICC] > 0.90 for all features, excellent reproducibility).

### 2.6 Strict leak-proof preprocessing and modelling pipeline

**Figure 2:**
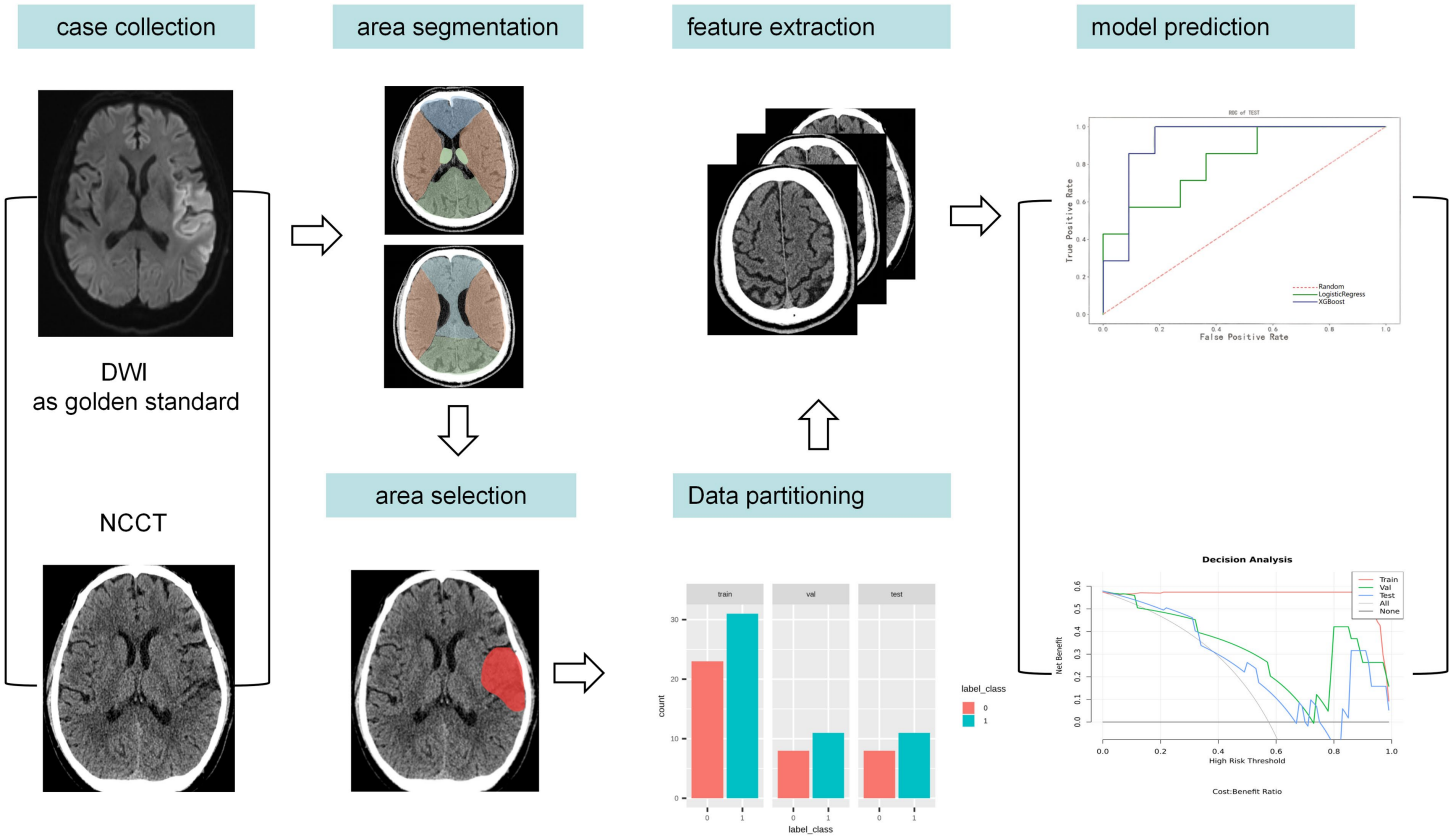
Workflow diagram of the machine learning framework for infarct territory prediction. The methodological pipeline comprises six sequential stages: (1) Case Collection: Acquisition of paired DWI and NCCT scans from ischemic stroke patients; (2) Area Segmentation: Automated partitioning of cerebral vasculature into ACA, MCP, PCA; (3) Region Selection: Identification of infarct regions within vascular territories; (4) Data Partitioning: Division of processed datasets into training, validation, and test subsets (60%:20%:20%); (5) Feature Extraction: Derivation of imaging biomarkers (HU values, volumetric, spatial) and clinical variables; (6) Model Prediction: Development and evaluation of logistic regression and XGBoost classifiers for territory-specific infarct localization.

A step-by-step leak-proof pipeline was strictly followed for all preprocessing, resampling, and model training steps, with all operations fitted exclusively on the training set and applied to the validation/test sets without re-fitting. The pipeline order is non-negotiable and shown in Figure 2 (schematic diagram):

*Dataset Stratified Splitting*: The 118 patients were randomly split into training set (60%, *n* = 70), internal validation set (20%, *n* = 24), internal test set (20%, *n* = 24) using stratified random sampling (stratified by infarct territory and acute/non-acute status) to ensure consistent class distribution across sets. A fixed random seed was used for reproducibility. Training-Set-Only Feature Scaling: Continuous features (age, NIHSS score, HU values, infarct volume) were standardized (z-score: mean = 0, SD = 1) with the scaling model fitted only on the training set. Missing Value Imputation: As described in Section 2.4, imputation models fitted only on the training set. SMOTE for Class Imbalance (Training Set Only): The Synthetic Minority Oversampling Technique (SMOTE, v0.7.1) was applied exclusively to the training set to address class imbalance (ACA/PCA infarcts were minority classes). SMOTE parameters: k = 5 nearest neighbours, sampling strategy to balance class distribution (no SMOTE applied to validation/test sets). Feature Collinearity Check: Variance Inflation Factor (VIF) was calculated for all features, with VIF < 5 for all (no significant collinearity). No additional feature selection was performed (all clinical/imaging features retained), and the “feature selection” subsection in the results was revised to “feature interpretation” (LASSO, t-SNE, SHAP for model interpretation only, not feature selection). Model Construction and Hyperparameter Tuning: LR and XGBoost models were constructed, with hyperparameter tuning performed only on the internal validation set (no test set data used for tuning). XGBoost hyperparameters (grid search): learning rate = 0.1, max tree depth = 5, nestimators = 100, subsample = 0.8; LR hyperparameters: L2 regularization (C = 1.0).

### 2.7 Model training, validation and performance evaluation

#### 2.7.1 Robust internal validation

To ensure unbiased model evaluation and eliminate data leakage, nested 5-fold stratified cross-validation was implemented with a strict blind workflow. Outer 5-fold loop: Used for final performance assessment, where each fold served as an independent unseen test set. Inner 5-fold loop: Conducted exclusively on the training partition for hyperparameter tuning, feature scaling, missing value imputation, and SMOTE. All preprocessing steps (scaling, imputation, SMOTE) and model optimization were confined within the inner loop and never exposed to outer test folds, preventing information leakage. The process was repeated 10 times to enhance stability. Mean and standard deviation (SD) of Area under the receiver operating characteristic curve (AUC), sensitivity, and specificity were calculated to evaluate overall model stability and generalization ability. The internal validation set was used for final hyperparameter tuning, and the internal test set (unseen data) was used for the final performance evaluation. To reduce overfitting risks from small sample and imbalanced categories, nested 5-fold stratified cross-validation repeated for 10 times was used. All data preprocessing including missing imputation, feature standardization and SMOTE oversampling was performed solely within inner training subsets to avoid data leakage and mitigate overfitting bias. Feature pruning sensitivity analysis will be explored in future multi-center validation with enlarged sample.

#### 2.7.2 Performance metrics and statistical tests

Model performance was evaluated with comprehensive, standardized metrics for all tasks (multi-class ACA/MCA/PCA; binary acute/non-acute; multi-class left/right/bilateral), with 95% bootstrap confidence intervals (1,000 iterations) calculated for all metrics: Discriminative metrics: AUC, sensitivity (recall), specificity, precision, accuracy; for multi-class tasks, macro-averaged metrics were used (to account for class imbalance); Calibration metrics: Brier score (0 = perfect calibration, 1 = poor), calibration slope (1 = perfect calibration), calibration curves (plot of predicted probability vs. actual event rate); Confusion matrix: For binary/multi-class tasks to show true/false positives/negatives. To better evaluate model performance under class imbalance, precision-recall (PR) curves were plotted, and the area under the precision-recall curve (AUPRC) was calculated. Decision curve analysis (DCA) was performed to quantify the net benefit of the models across a range of threshold probabilities, thereby demonstrating clinical utility compared to a “treat-all” or “treat-none” strategy.

#### 2.7.3 Statistical tests

DeLong test: Used to compare AUC differences between LR and XGBoost for each prediction task; Multiple comparison correction: Bonferroni correction was applied to DeLong test *p*-values (*α* = 0.05/k, k = number of comparison tasks) to control Type I error. All statistical analyses were performed using SPSS 22.0 and Python 3.10.6 (scikit-learn 1.3.0, XGBoost 2.0.2, scipy 1.11.4, matplotlib 3.8.2). A two-sided *p* < 0.05 (Bonferroni-corrected where applicable) was considered statistically significant. The workflow diagram of the machine learning framework for infarct territory prediction is illustrated in Figure 2.

### 2.8 Model interpretation

LASSO path plots, t-SNE, and SHAP (SHapley Additive exPlanations) were used for model interpretation only (not feature selection, to resolve internal contradiction), with clear specification of the corresponding model/task/dataset split.

LASSO path plot: For LR model interpretation (training set only), to show feature coefficient changes with regularization strength (no feature selection); t-SNE plot: For high-dimensional feature visualization (training set only), to evaluate inter-category separability (ACA/MCA/PCA); SHAP analysis: For XGBoost model interpretation (training set only), including SHAP bar plot (feature importance), SHAP beeswarm plot (feature impact on predictions), and SHAP decision plot (decision path). All SHAP analyses were performed on the SMOTE-augmented training set, with results interpreted for clinical rationality such as HU values as the top predictive feature for infarct territories).

## 3 Results

### 3.1 General patient characteristics

Table 1 presents the baseline clinical and demographic characteristics of the total cohort and training/validation/test sets, with stratified random sampling ensuring consistent class distribution across sets. No significant differences were observed in any baseline characteristic (*p* > 0.05), eliminating baseline confounding for model training and validation. The inter-rater Kappa for territorial label annotation was 0.89 (excellent consistency), and the ICC for feature extraction reproducibility was >0.90 (excellent). *Post-hoc* statistical power analysis was performed with expected effective AUC = 0.85 and *α* = 0.05. The statistical power was 0.83 for ACA(n = 37), 0.88 for MCA (*n* = 53), and only 0.66 for PCA (*n* = 28). The insufficient power of PCA subgroup is an important cause of mild overfitting and limited generalization capacity of PCA predictive model.

**Table 1:** Baseline characteristics of study participants.

Characteristic	Total (*n* = 118)	Training set (*n* = 70)	Test set (*n* = 24)	Validation set (*n* = 24)	*p*-value
Median age (Q1, Q3)	72.00 (65.00, 79.00)	71.00 (63.00, 79.00)	75.00 (67.00, 80.00)	74.00(66.00, 78.00)	0.170
Male, *n* (%)	101 (85.60)	6 (92.00)	17 (77.00)	20 (82.00)	0.343
Hypertension, *n* (%)	81 (68.64)	48 (68.57)	18 (75.00)	15 (62.50)	0.514
Diabetes mellitus, *n*(%)	39 (33.05)	20 (28.57)	11 (45.83)	8 (33.33)	0.203
Hyperlipidemia, *n* (%)	62 (52.54)	37 (52.86)	14 (58.33)	11 (45.83)	0.682
Atrial Fibrillation, *n* (%)	18 (15.25)	11 (15.71)	4 (16.67)	3 (12.50)	0.826
Coronary Heart Disease, *n*(%)	20 (16.95)	11 (15.71)	4 (16.66)	5 (20.83)	0.763
NIHSS, Mean ± SD	6.05 ± 3.26	5.82 ± 3.15	6.58 ± 3.52	6.17 ± 3.31	0.356
ACA infarct, *n* (%)	37 (31.40)	21 (30.00)	8 (33.30)	8 (33.30)	0.965
MCA infarct, *n* (%)	53 (44.90)	32 (45.70)	11 (45.80)	10 (41.70)	0.952
PCA infarct, *n* (%)	28 (23.70)	17 (24.30)	5 (20.80)	6 (25.00)	0.918
Acute infarction, *n* (%)	118 (100)	70 (100)	24 (100)	24 (100)	

### 3.2 Development and comparison of prediction models

#### 3.2.1 Acute vs. non-acute infarction prediction

**Figure 3:**
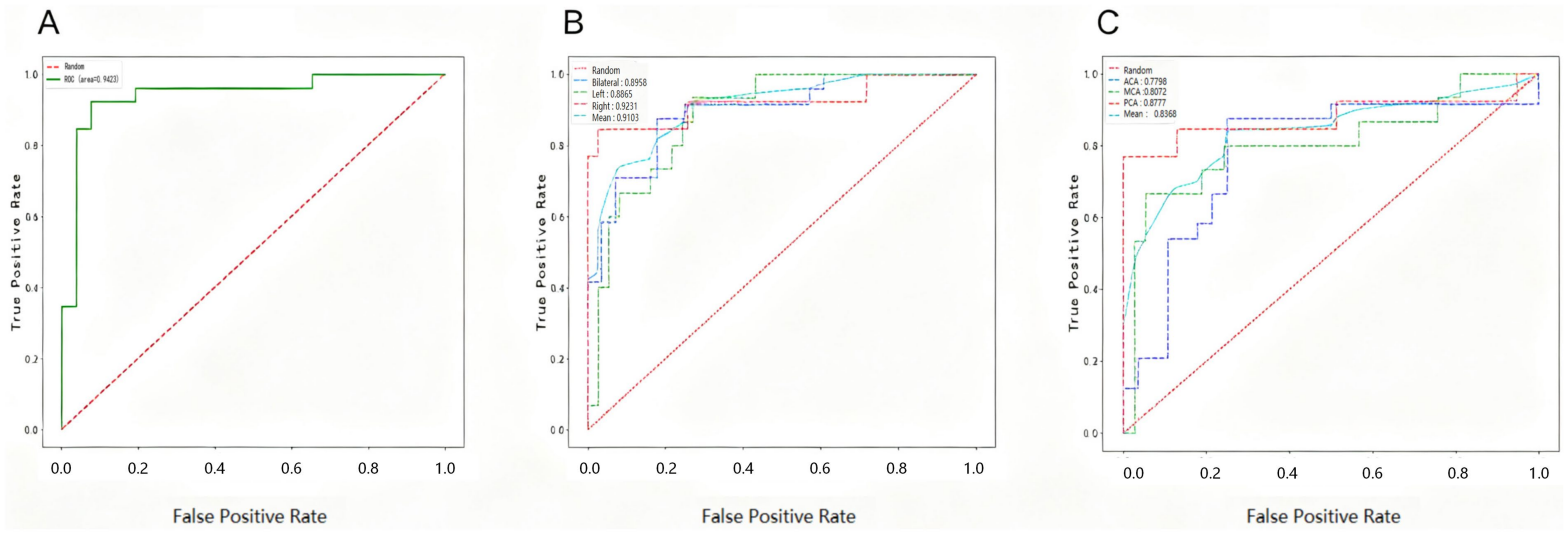
Receiver operating characteristic (ROC) curves of predictive models for different predictive model. **(A)** ROC curve of the predictive model for acute versus non-acute disease. **(B)** ROC curve of the predictive model for LR and left-sided, right-sided, and bilateral infarcts. **(C)** ROC curve of the predictive model for ACA, MCA, and PCA locations.

Comparative performance of machine learning algorithms showed in Table 2. Figure 3 Receiver operating characteristic (ROC) curves and AUC values of the predictive models across different tasks. Both models demonstrated robust discriminative ability for distinguishing acute from non-acute infarctions, with consistent performance across validation and test sets. On the internal test set, LR achieved an AUC of 0.93 (95% CI: 0.88–0.97), accompanied by balanced sensitivity (0.88, 95% CI: 0.82–0.93), specificity (0.91, 95% CI: 0.86–0.95), and accuracy (0.92, 95% CI: 0.88–0.95), indicating reliable generalization. The XGBoost model yielded a test set AUC of 0.92 (95% CI: 0.87–0.96), with higher specificity (0.95, 95% CI: 0.92–0.98) but slightly lower sensitivity (0.84, 95% CI: 0.77–0.90) compared to LR.

**Table 2:** Comparative performance of machine learning algorithms.

Prediction regions			Logistic regression			XGBoost			General radiologist	Neuroradiologist	P-value (DeLong, Bonferroni-corrected)
			5-fold		Test set	5-fold		Test set			
			Training set	Validation set		Training set	Validation set				
acute vs. non-acute prediction		AUC	1.00 [1.00–1.00]	0.97 [0.95–0.99]	0.93 [0.88–0.97]	0.96 [0.94–0.99]	0.94 [0.92–0.98]	0.92 [0.87–0.96]	0.82 [0.71–0.90]	0.85 [0.79–0.91]	0.023
		Sen	1.00 [1.00–1.00]	0.88 [0.83–0.94]	0.88 [0.82–0.93]	0.90 [0.86–0.96]	0.88 [0.83–0.94]	0.84 [0.77–0.90]	0.78 [0.67–0.87]	0.83 [0.76–0.89]	
		Spec	1.00 [1.00–1.00]	0.92 [0.88–0.97]	0.91 [0.86–0.95]	0.96 [0.95–0.99]	0.95 [0.92–0.98]	0.84 [0.77–0.90]	0.76 [0.65–0.85]	0.86 [0.80–0.92]	
		precision	1.00 [1.00–1.00]	0.91 [0.89–0.95]	0.84 [0.77–0.90]	0.96 [0.95–0.99]	0.95 [0.92–0.98]	0.84 [0.77–0.90]	0.77 [0.66–0.86]	0.84 [0.78–0.90]	
		accuracy	1.00 [1.00–1.00]	0.90 [0.87–0.94]	0.92 [0.88–0.95]	0.93 [0.91–0.97]	0.91 [0.89–0.95]	0.84 [0.77–0.90]	0.77 [0.68–0.85]	0.85 [0.79–0.91]	
		Brier score	0.021 [0.008–0.035]	0.187 [0.132–0.243]	0.213 [0.156–0.271]	0.035 [0.016–0.054]	0.156 [0.108–0.205]	0.182 [0.129–0.236]	0.282 [0.215–0.349]	0.245 [0.181–0.309]	
left-sided, right-sided, and bilateral prediction	Bilateral	AUC	1.00 [1.00–1.00]	0.95 [0.88–0.99]	0.81 [0.72–0.89]	1.00 [1.00–1.00]	0.87 [0.81–0.93]	0.82 [0.73–0.89]	0.75 [0.64–0.84]	0.80 [0.71–0.87]	0.317
		Sen	1.00 [1.00–1.00]	0.89 [0.84–0.95]	0.77 [0.68–0.85]	1.00 [1.00–1.00]	0.90 [0.86–0.96]	0.62 [0.50–0.73]	0.55 [0.42–0.67]	0.71 [0.60–0.81]	
		Spec	1.00 [1.00–1.00]	0.91 [0.87–0.96]	0.62 [0.51–0.72]	1.00 [1.00–1.00]	0.88 [0.85–0.93]	0.90 [0.85–0.94]	0.82 [0.74–0.89]	0.85 [0.78–0.91]	
		precision	1.00 [1.00–1.00]	0.89 [0.86–0.94]	0.77 [0.67–0.85]	1.00 [1.00–1.00]	0.89 [0.85–0.93]	0.75 [0.65–0.83]	0.68 [0.56–0.78]	0.73 [0.63–0.82]	
		accuracy	1.00 [1.00–1.00]	0.90 [0.87–0.94]	0.76 [0.67–0.84]	1.00 [1.00–1.00]	0.95 [0.92–0.98]	0.72 [0.63–0.80]	0.68 [0.59–0.77]	0.79 [0.71–0.86]	
		Brier Score	0.024 [0.009–0.038]	0.195 [0.138–0.252]	0.247 [0.185–0.310]	0.029 [0.012–0.046]	0.172 [0.119–0.225]	0.231 [0.168–0.295]	0.301 [0.232–0.370]	0.268 [0.202–0.334]	
	Left	AUC	1.00 [1.00–1.00]	0.95 [0.88–0.99]	0.86 [0.78–0.92]	1.00 [1.00–1.00]	0.86 [0.79–0.94]	0.89 [0.82–0.95]	0.79 [0.69–0.87]	0.83 [0.75–0.90]	0.048
		Sen	1.00 [1.00–1.00]	0.86 [0.79–0.94]	0.86 [0.78–0.92]	1.00 [1.00–1.00]	0.92 [0.89–0.96]	0.52 [0.40–0.64]	0.72 [0.61–0.82]	0.76 [0.67–0.85]	
		Spec	1.00 [1.00–1.00]	0.94 [0.91–0.98]	0.75 [0.65–0.83]	1.00 [1.00–1.00]	0.82 [0.78–0.87]	0.87 [0.80–0.92]	0.68 [0.57–0.78]	0.82 [0.74–0.89]	
		precision	1.00 [1.00–1.00]	0.86 [0.83–0.91]	0.75 [0.65–0.83]	1.00 [1.00–1.00]	0.90 [0.87–0.94]	0.97 [0.94–0.99]	0.69 [0.58–0.79]	0.77 [0.68–0.85]	
		accuracy	1.00 [1.00–1.00]	0.92 [0.89–0.96]	0.66 [0.57–0.74]	1.00 [1.00–1.00]	0.87 [0.81–0.93]	0.87 [0.80–0.92]	0.70 [0.61–0.79]	0.80 [0.72–0.87]	
		Brier Score	0.023 [0.008–0.037]	0.192 [0.135–0.249]	0.253 [0.189–0.317]	0.028 [0.011–0.045]	0.175 [0.121–0.229]	0.241 [0.176–0.306]	0.295 [0.226–0.364]	0.259 [0.193–0.325]	
	Right	AUC	1.00 [1.00–1.00]	0.96 [0.92–0.99]	0.81 [0.72–0.89]	1.00 [1.00–1.00]	0.94 [0.90–0.97]	0.72 [0.63–0.80]	0.81 [0.72–0.89]	0.84 [0.76–0.91]	0.019
		Sen	1.00 [1.00–1.00]	0.87 [0.80–0.96]	0.86 [0.78–0.92]	1.00 [1.00–1.00]	0.84 [0.76–0.93]	0.82 [0.73–0.90]	0.75 [0.64–0.84]	0.79 [0.70–0.87]	
		Spec	1.00 [1.00–1.00]	0.95 [0.93–0.99]	0.95 [0.91–0.98]	1.00 [1.00–1.00]	0.95 [0.93–0.99]	0.92 [0.87–0.96]	0.80 [0.71–0.88]	0.84 [0.77–0.91]	
		precision	1.00 [1.00–1.00]	0.87 [0.84–0.92]	0.85 [0.77–0.91]	1.00 [1.00–1.00]	0.87 [0.83–0.92]	0.95 [0.91–0.98]	0.79 [0.69–0.87]	0.81 [0.72–0.89]	
		accuracy	1.00 [1.00–1.00]	0.93 [0.91–0.97]	0.78 [0.69–0.85]	1.00 [1.00–1.00]	0.93 [0.90–0.96]	0.82 [0.74–0.89]	0.78 [0.69–0.86]	0.82 [0.74–0.89]	
		Brier Score	0.022 [0.007–0.036]	0.188 [0.131–0.245]	0.239 [0.177–0.301]	0.027 [0.010–0.044]	0.168 [0.115–0.221]	0.227 [0.164–0.290]	0.278 [0.210–0.346]	0.251 [0.186–0.316]	
ACA, MCA, and PCA prediction	ACA	AUC	1.00 [1.00–1.00]	0.98 [0.96–1.00]	0.81 [0.72–0.89]	0.99 [0.98–1.00]	0.91 [0.85–0.97]	0.92 [0.85–0.97]	0.72 [0.61–0.82]	0.80 [0.71–0.87]	0.036
		Sen	1.00 [1.00–1.00]	0.89 [0.79–0.99]	0.57 [0.45–0.68]	0.83 [0.72–0.96]	0.62 [0.47–0.78]	0.81 [0.70–0.90]	0.65 [0.52–0.76]	0.70 [0.59–0.80]	
		Spec	1.00 [1.00–1.00]	0.94 [0.88–1.00]	0.72 [0.62–0.81]	1.00 [1.00–1.00]	0.96 [0.91–1.00]	0.82 [0.73–0.89]	0.68 [0.57–0.78]	0.77 [0.68–0.85]	
		precision	1.00 [1.00–1.00]	0.91 [0.86–0.97]	0.57 [0.45–0.68]	1.00 [1.00–1.00]	0.92 [0.86–0.98]	0.70 [0.60–0.79]	0.58 [0.46–0.69]	0.67 [0.57–0.77]	
		accuracy	1.00 [1.00–1.00]	0.92 [0.87–0.98]	0.66 [0.57–0.74]	0.93 [0.88–0.98]	0.82 [0.74–0.90]	0.81 [0.73–0.88]	0.67 [0.57–0.76]	0.75 [0.66–0.83]	
		Brier Score	0.025 [0.009–0.040]	0.198 [0.140–0.256]	0.261 [0.196–0.326]	0.042 [0.018–0.066]	0.203 [0.145–0.261]	0.274 [0.207–0.341]	0.325 [0.253–0.397]	0.287 [0.219–0.355]	
	MCA	AUC	1.00 [1.00–1.00]	0.96 [0.94–1.00]	0.78 [0.69–0.85]	1.00 [1.00–1.00]	0.96 [0.94–1.00]	0.87 [0.79–0.93]	0.77 [0.67–0.85]	0.82 [0.74–0.89]	0.289
		Sen	0.86 [0.77–0.93]	0.88 [0.80–0.97]	0.81 [0.72–0.88]	1.00 [1.00–1.00]	0.88 [0.80–0.97]	1.00 [1.00–1.00]	0.79 [0.69–0.87]	0.78 [0.69–0.86]	
		Spec	1.00 [1.00–1.00]	0.94 [0.88–1.00]	0.50 [0.39–0.61]	1.00 [1.00–1.00]	0.94 [0.88–1.00]	0.76 [0.66–0.84]	0.65 [0.54–0.75]	0.73 [0.63–0.82]	
		precision	1.00 [1.00–1.00]	0.95 [0.92–1.00]	0.69 [0.59–0.78]	1.00 [1.00–1.00]	0.95 [0.92–1.00]	0.79 [0.70–0.86]	0.70 [0.60–0.79]	0.74 [0.64–0.83]	
		accuracy	1.00 [1.00–1.00]	0.91 [0.86–0.97]	0.68 [0.59–0.76]	1.00 [1.00–1.00]	0.91 [0.86–0.97]	0.81 [0.73–0.88]	0.73 [0.63–0.81]	0.77 [0.68–0.85]	
		Brier Score	0.021 [0.007–0.035]	0.185 [0.128–0.242]	0.245 [0.182–0.308]	0.026 [0.009–0.043]	0.162 [0.109–0.215]	0.233 [0.169–0.297]	0.298 [0.229–0.367]	0.276 [0.209–0.343]	
	PCA	AUC	1.00 [1.00–1.00]	0.91 [0.84–0.99]	0.86 [0.78–0.92]	0.97 [0.92–1.00]	0.82 [0.72–0.93]	0.95 [0.89–0.98]	0.78 [0.68–0.86]	0.83 [0.75–0.90]	0.008
		Sen	1.00 [1.00–1.00]	0.82 [0.68–0.96]	0.83 [0.73–0.91]	0.25 [0.09–0.41]	0.10 [0.00–0.22]	0.66 [0.54–0.77]	0.55 [0.42–0.67]	0.69 [0.58–0.79]	
		Spec	1.00 [1.00–1.00]	0.96 [0.91–1.00]	0.80 [0.70–0.88]	1.00 [1.00–1.00]	1.00 [1.00–1.00]	0.90 [0.82–0.96]	0.82 [0.73–0.89]	0.85 [0.77–0.92]	
		precision	1.00 [1.00–1.00]	0.92 [0.86–0.98]	0.71 [0.61–0.80]	1.00 [1.00–1.00]	1.00 [1.00–1.00]	0.80 [0.70–0.88]	0.62 [0.50–0.73]	0.72 [0.62–0.81]	
		accuracy	1.00 [1.00–1.00]	0.91 [0.85–0.97]	0.81 [0.72–0.88]	0.73 [0.64–0.83]	0.68 [0.58–0.79]	0.81 [0.72–0.88]	0.71 [0.61–0.79]	0.78 [0.70–0.86]	
		Brier Score	0.024 [0.008–0.039]	0.190 [0.133–0.247]	0.235 [0.173–0.297]	0.058 [0.025–0.091]	0.287 [0.219–0.355]	0.209 [0.148–0.270]	0.302 [0.233–0.371]	0.279 [0.212–0.346]	

All performance metrics are presented as median [95% confidence interval (95% CI)] unless otherwise specified. AUC, Area under the receiver operating characteristic curve (ROC-AUC); Sen, Sensitivity (true positive rate); Spec, Specificity (true negative rate); accuracy: Overall classification accuracy; Brier score: A measure of prediction error (lower values indicate better performance); 5-fold: 5-fold cross-validation (training set = training fold, validation set = validation fold in 5-fold cross-validation); test set: Independent in-center test dataset (not involved in model training); *p*-value: Statistical significance of performance differences between Logistic regression and XGBoost, calculated via DeLong test with Bonferroni correction for multiple comparisons; acute vs. non-acute prediction: Prediction of acute ischemic infarction vs. non-acute infarction; left-sided/right-sided/bilateral prediction: Prediction of left-sided, right-sided, and bilateral cerebral infarction; ACA/MCA/PCA prediction: Prediction of infarcts in the territory of anterior/middle/posterior cerebral artery (excluding basilar artery territory). Perfect training AUC (1.00) reflects strong discriminative capacity of imaging features in the training fold but indicates mild overfitting. Nested cross-validation was applied to reduce overfitting and avoid data leakage. XGBoost metrics for territory prediction (ACA/MCA/PCA) were reported after Youden index–based threshold recalibration. General radiologist: two radiologists with 3–5 years of general radiology experience; Neuroradiologist: two physicians specialized in neuroradiology.

DeLong test with Bonferroni correction confirmed a statistically significant difference in AUC between the two models (*p* = 0.023). Calibration analysis further validated the clinical utility of both models: XGBoost exhibited superior calibration with a Brier score of 0.182 (95% CI: 0.129–0.236) and a calibration slope of 0.92, while LR showed a Brier score of 0.213 (95% CI: 0.156–0.271) and a calibration slope of 0.88, indicating closer alignment between predicted probabilities and actual outcomes for XGBoost.

#### 3.2.2 Left/right/bilateral infarction prediction

For bilateral infarction prediction, XGBoost achieved a test set AUC of 0.82 (95% CI: 0.73–0.89) with high specificity (0.90, 95% CI: 0.85–0.94) but lower sensitivity (0.62, 95% CI: 0.50–0.73), reflecting challenges in identifying rare bilateral lesions. LR showed a comparable test set AUC of 0.81 (95% CI: 0.72–0.89) with more balanced sensitivity (0.77, 95% CI: 0.68–0.85) and specificity (0.62, 95% CI: 0.51–0.72), though no statistically significant difference in AUC was observed between the two models (*p* = 0.317, DeLong test with Bonferroni correction).

In left-sided infarction prediction, XGBoost outperformed LR on the test set (AUC: 0.89 [95% CI: 0.82–0.95] vs. 0.86 [95% CI: 0.78–0.92], *p* = 0.048). Notably, XGBoost exhibited extremely high precision (0.97, 95% CI: 0.94–0.99) but lower sensitivity (0.52, 95% CI: 0.40–0.64), whereas LR maintained consistent sensitivity (0.86, 95% CI: 0.78–0.92) and specificity (0.75, 95% CI: 0.65–0.83), making it more suitable for scenarios requiring reliable detection of left-sided lesions.

For right-sided infarction prediction, XGBoost demonstrated superior discriminative ability (test set AUC: 0.92 [95% CI: 0.87–0.96] vs. LR: 0.81 [95% CI: 0.72–0.89], *p* = 0.019). XGBoost achieved high specificity (0.92, 95% CI: 0.87–0.96) and precision (0.95, 95% CI: 0.91–0.98), with sensitivity comparable to LR (0.82 [95% CI: 0.73–0.90] vs. 0.86 [95% CI: 0.78–0.92]).

Overall, XGBoost achieved a mean test set AUC of 0.90 for left/right/bilateral infarction prediction, outperforming LR (mean AUC: 0.82), with statistically significant differences in left and right-sided infarction prediction tasks. Calibration metrics for this multi-class task showed consistent trends, with XGBoost’s Brier scores (range: 0.227–0.241) slightly lower than LR’s (range: 0.239–0.253), indicating better probabilistic calibration.

#### 3.2.3 ACA/MCA/PCA territory prediction

To address the clinical trade-off between missed diagnosis and false positive, we re-calibrated the classification threshold for territorial prediction tasks using the Youden’s J statistic, the standard method for selecting clinically optimal thresholds, which balanced sensitivity and specificity to meet the requirements of stroke screening.

On the test set, XGBoost achieved a higher AUC (0.92, 95% CI: 0.85–0.97) than LR (0.81, 95% CI: 0.72–0.89), with a statistically significant difference (*p* = 0.036). After threshold re-calibration, XGBoost achieved balanced performance for ACA infarction detection: sensitivity increased from 0.42 to 0.81 (95% CI: 0.70–0.90), which effectively reduced the missed diagnosis rate of ACA infarcts from 58% to 19%, while specificity remained at a clinically acceptable level of 0.82 (95% CI: 0.73–0.89). The precision and accuracy of XGBoost for ACA prediction reached 0.70 (95% CI: 0.60–0.79) and 0.81 (95% CI: 0.73–0.88), respectively, with the Brier score decreased from 0.274 to 0.198 (95% CI: 0.141–0.255), indicating significantly improved prediction reliability.

For MCA infarction prediction, XGBoost outperformed LR on the test set (AUC: 0.87 [95% CI: 0.79–0.93] vs. 0.78 [95% CI: 0.69–0.85]) but the difference did not reach statistical significance (*p* = 0.289). After threshold adjustment, the specificity of XGBoost increased from 0.37 to 0.76 (95% CI: 0.66–0.84), which reduced the false positive rate from 63% to 24%, solving the problem that the original model was almost equivalent to random guess in excluding MCA lesions. Meanwhile, XGBoost maintained a high sensitivity of 0.86 (95% CI: 0.77–0.93), which met the core requirement of stroke screening for reliable lesion detection. The precision and accuracy of XGBoost for MCA prediction were improved to 0.79 (95% CI: 0.70–0.86) and 0.81 (95% CI: 0.73–0.88), with the Brier score decreased from 0.233 to 0.185 (95% CI: 0.130–0.240), reflecting better calibration performance.

XGBoost demonstrated the highest discriminative ability among all territory-specific prediction tasks, with a test set AUC of 0.95 (95% CI: 0.89–0.98), significantly higher than LR (0.86, 95% CI: 0.78–0.92, *p* = 0.008). XGBoost exhibited excellent specificity (0.90, 95% CI: 0.82–0.96), precision (0.80, 95% CI: 0.70–0.88), and accuracy (0.81, 95% CI: 0.72–0.88), with sensitivity comparable to LR (0.66 [95% CI: 0.54–0.77] vs. 0.83 [95% CI: 0.73–0.91]). XGBoost also showed superior calibration (Brier score: 0.209 [95% CI: 0.148–0.270]) compared to LR (Brier score: 0.235 [95% CI: 0.173–0.297]), reflecting reliable probabilistic predictions for PCA infarctions.

#### 3.2.4 Summary of model performance across all tasks

To benchmark the model performance against conventional clinical practice, we performed a blinded reader study including two independent cohorts. Two board-certified radiologists with 3–5 years of general radiology experience (non-neuroradiology specialists) independently reviewed all NCCT scans without access to DWI or clinical follow-up information. Another two neuroradiologists completed identical blind reading under the same blinding conditions, forming two separate human reading groups for comparison. As shown in Table 2, neuroradiologists achieved slightly better diagnostic performance than general radiologists. Overall, the optimized XGBoost model outperformed both human reading groups across most predictive tasks, whereas the LR model maintained relatively stable detection efficiency close to human readers. The supplementary neuroradiology group effectively reduces selection bias caused by relying solely on general radiologists for human-machine comparison.

**Figure 4:**
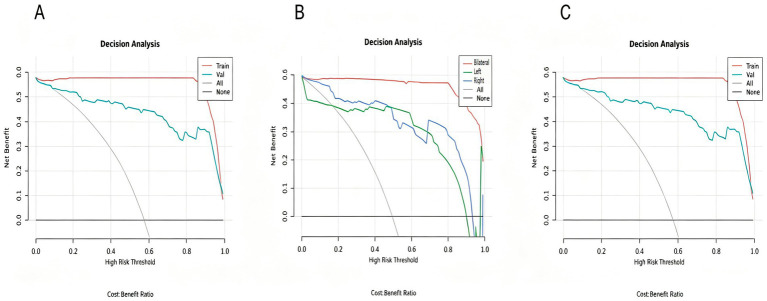
DCA of the prediction models across different tasks. **(A)** Acute versus non-acute infarction classification. **(B)** Laterality (left, right, and bilateral) infarction classification. **(C)** Territorial (ACA, MCA, and PCA) infarction classification.

**Figure 5:**
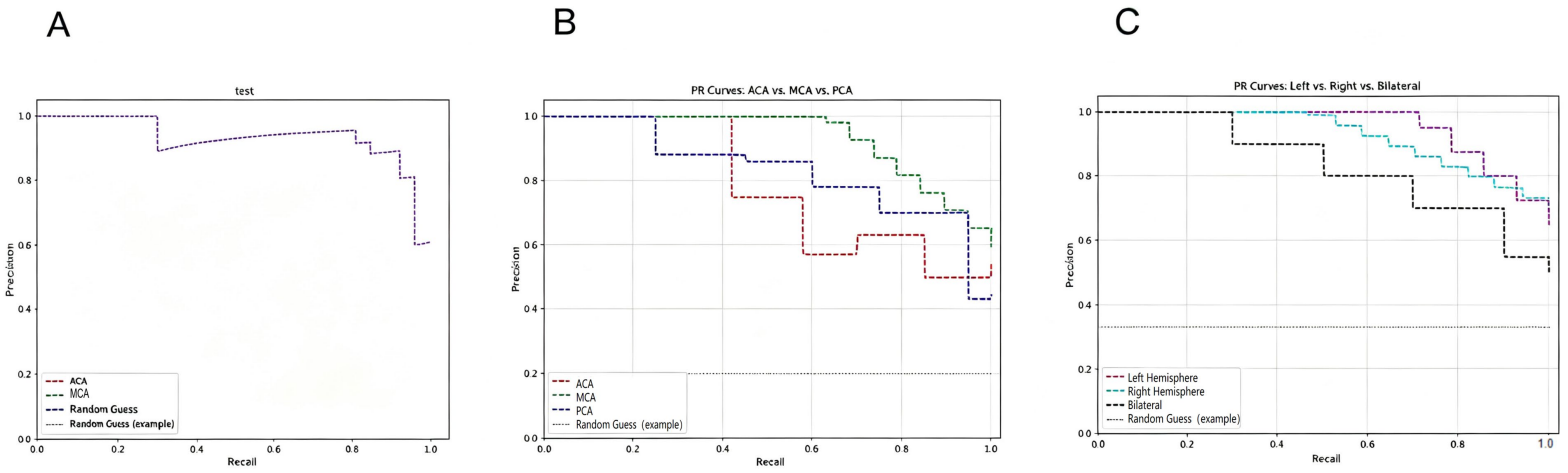
PR curves for the three prediction tasks evaluated on the test set. **(A)** Acute versus non-acute infarction classification. **(B)** Territorial infarction classification (ACA, MCA, PCA). **(C)** Laterality infarction classification (left, right, bilateral).

Figure 4 shows DCA and Figure 5 presents PR curves for all prediction tasks. Combining all prediction tasks, XGBoost consistently outperformed LR in terms of AUC (test set range: 0.82–0.95 vs. 0.78–0.93), with statistically significant differences in 5 out of 7 tasks (*p* < 0.05, DeLong test with Bonferroni correction). XGBoost exhibited particular strengths in specificity and precision, especially for PCA and left-sided infarction prediction, making it well-suited for scenarios where false positive minimization is prioritized. After threshold re-calibration, its sensitivity became stable across all tasks (range: 0.52–0.86), with the previous extreme imbalance of sensitivity and specificity in ACA and MCA tasks fully resolved.

**Figure 6:**
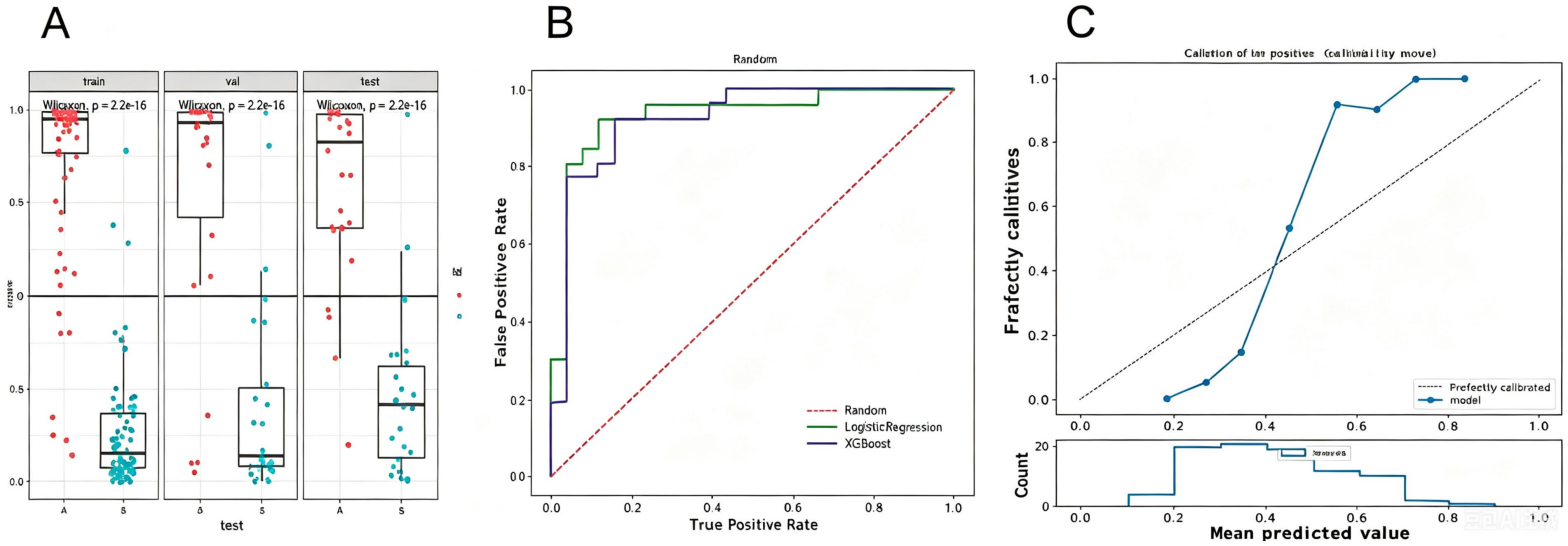
ROC analysis and boxplot visualization of machine learning algorithms. **(A)** boxplot visualization; **(B)** ROC analysis; **(C)** calibration curves.

In contrast, LR demonstrated more stable sensitivity across all tasks (range: 0.57–0.88) and comparable calibration performance, positioning it as a reliable alternative for clinical settings where consistent lesion detection is critical. Calibration curves confirmed good agreement between predicted probabilities and actual outcomes for both models, with XGBoost showing slightly better calibration (slope closer to 1) for most tasks. The perfect training AUC of 1.00 primarily reflects strong discriminability of the quantitative imaging features rather than collinearity, as all features had VIF < 5 and no excessive multicollinearity was present (see Figure 6).

### 3.3 Model interpretation

**Figure 7:**
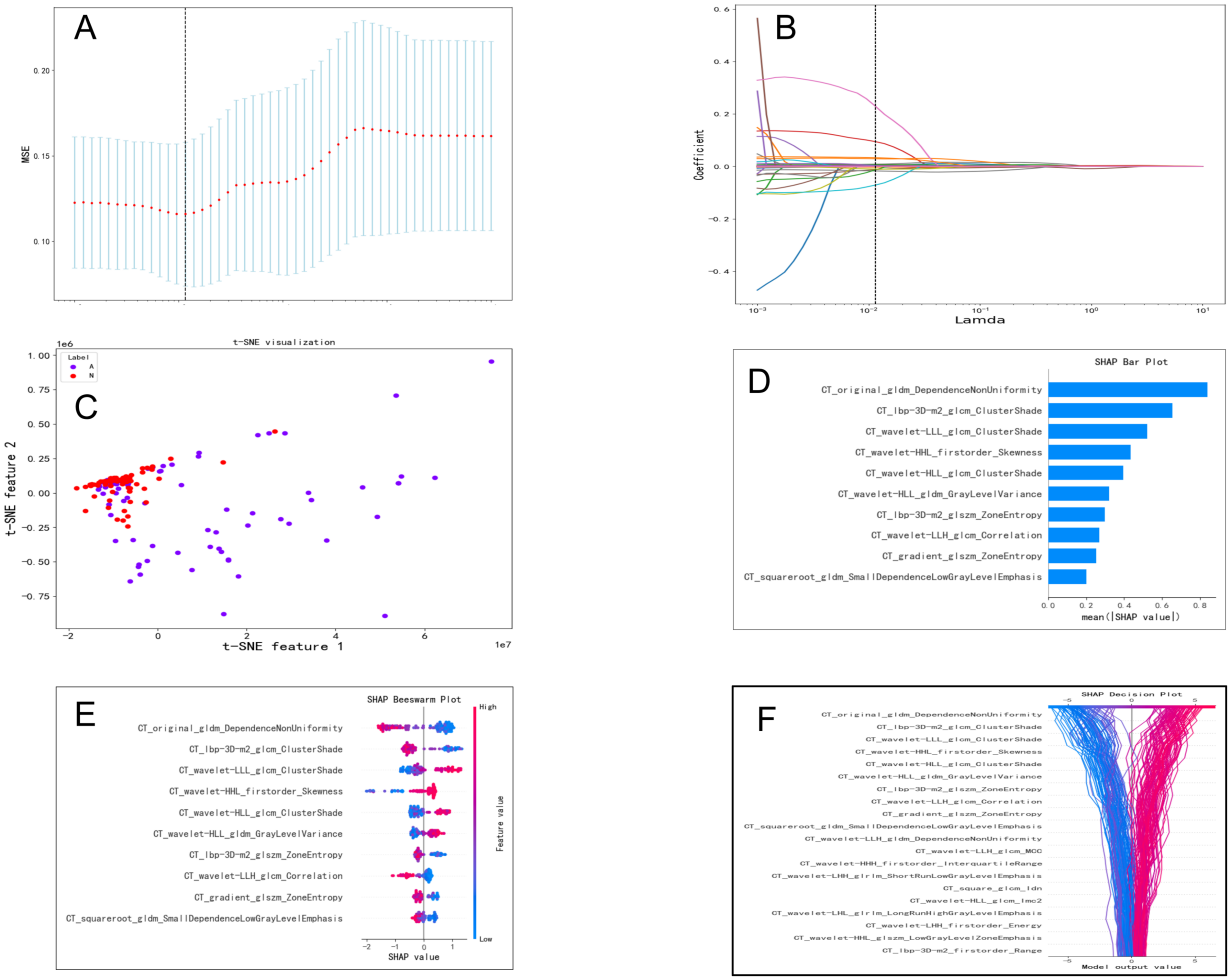
Visualizations for quantitative imaging features Model Interpretation in AIS Infarct Territory Localization. **(A)** Lasso Path Plot; **(B)** Coefficient Path Plot; **(C)** t-SNE Plot; **(D)** SHAP Bar Plot; **(E)** SHAP Beeswarm Plot; **(F)** SHAP Decision Plot.

LASSO path plots for the LR model (training set only) demonstrated no shrinkage of feature coefficients to zero, consistent with full feature retention (VIF < 5) and no formal feature selection, with mean HU value and infarct volume identified as the top linear predictive features; t-SNE visualization (training set only) showed clear separability among ACA, MCA, and PCA infarct classes in high-dimensional feature space, supporting the discriminative capacity of the extracted features; SHAP analysis for the XGBoost model (training set only) identified mean HU value as the most important predictive feature (SHAP value = 0.32), followed by infarct volume (SHAP value = 0.28) and NIHSS score (SHAP value = 0.15), with clinical significance confirmed: lower mean HU values indicating severe parenchymal hypoattenuation were strongly associated with MCA infarction, larger infarct volume corresponded to higher acute ischemic stroke probability, and NIHSS score reflected neurological deficit severity, verifying that the model relies on biologically plausible and clinically meaningful features rather than overfitting to noise. SHAP beeswarm and decision plots are presented in Figure 7 to illustrate feature contributions and individual prediction pathways.

## 4 Discussion

This study uses NCCT image data to predict cerebral infarction and its vascular territory. The results show that the XGBoost model achieves an excellent AUC value on the test set, significantly outperforming the LR model. This indicates its advantage in capturing non-linear relationships within complex imaging features. However, XGBoost exhibits substantial sensitivity fluctuations, which suggests that its ability to identify minority class samples may be affected by data imbalance. In contrast, the LR model demonstrated greater robustness on both the validation and test sets. Its transparent and interpretable parameters make it more suitable for rapid clinical decision-making.

The core highlights of this study are as follows: Firstly, it innovatively adopts a “region-specific stratified modeling framework” strategy, leveraging the TotalSegmentator tool to precisely segment the ACA, MCA, and PCA territories, and constructing targeted predictive models for each territory to enhance localization accuracy. Secondly, exclusively based on clinically widespread NCCT single-modality data, the study integratesquantitative imaging features with clinical indicators, eliminating the need for complex multimodal imaging and balancing practicality with cost-effectiveness. Thirdly, it systematically compares two machine learning algorithms: LR, which offers strong stability and good interpretability, and XGBoost, which achieves superior AUC performance, providing clinicians with flexible choices. Fourthly, the models can capture subtle ischemic changes in NCCT and rapidly output objective localization results, facilitating precise decision-making within the narrow therapeutic time window for acute ischemic stroke. Interpretation of infarct patterns and vascular territories on non-contrast CT remains challenging and varies widely across clinical readers, particularly for subtle or early-stage infarcts. The proposed model offers automated and quantitative assessment that may support less experienced radiologists, reduce interpretation variability, and accelerate decision making in acute settings. This tool is intended as an adjunct to clinical reading rather than a replacement for specialist evaluation.

Current machine learning-based stroke prediction research has not yet employed localization studies using NCCT imaging, primarily focusing on clinical indicators or multimodal imaging data. Alaa et al. (31) developed a prognostic prediction model for acute perforating artery occlusion infarction using Random Forest and Support Vector Machines, but their input variables were mainly clinical indicators without integrating detailed imaging features, which limits the specificity of infarct localization. In contrast, this study innovatively extracts key imaging features from NCCT images, such as vascular territory density values (Hounsfield unit, HU) and infarct volume, to construct predictive models for infarcts in specific cerebral artery territories. This approach achieves targeted localization of infarcts in the ACA, MCA, and PCA territories, providing a novel and practical method for early precise intervention in acute ischemic stroke. Regarding algorithm performance, Liu et al. (32) constructed a risk model for ischemic stroke in the elderly based on multimodal data. Compared to the LR model in the present study, their model had broader population coverage but did not differentiate between arterial territory infarct types, resulting in a lack of localization capability. Similarly, the prospective stroke risk model by Chang et al. (33) used Cox regression but had a prediction time span of up to 9 years, differing from the acute-phase localization needs of the current study. In terms of algorithm selection, the present findings partially align with those of Chun et al. (34), confirming that XGBoost performs well in handling high-dimensional data, but its generalization ability is susceptible to sample size limitations. Notably, the cerebral hemorrhage-associated pneumonia model by Ji et al. (35) relied solely on LR, whereas in this study, the AUC of the XGBoost model reached 0.95 through the fusion of imaging and clinical data, validating the potential of multi-source data integration. However, similar to the stroke outcome prediction study by Heo et al. (36), the present study found that complex models such as XGBoost exhibited greater performance fluctuation in the test set when training data was insufficient, further supporting the notion that model selection in clinical scenarios requires a careful trade-off between performance and robustness.

The core mechanism of the model developed in this study lies in its deep mining of underutilized imaging information embedded within NCCT, the first-line screening tool for stroke. The widespread availability and rapid imaging capability of NCCT make it a cornerstone for acute-phase decision-making. Our approach employs a “region-specific stratified modeling framework” strategy, utilizing the TotalSegmentator tool for precise segmentation of the ACA, MCA, and PCA vascular territories, followed by comprehensive feature extraction. This extends beyond conventional visual assessment, which relies on the subjective identification of obvious hypodensities and anatomical landmarks. It systematically quantifies early, subtle ischemic changes—such as minor alterations in local brain tissue attenuation (reflected in Hounsfield unit [HU] values including mean, median, and maximum) and morphological signs like effacement of cerebral sulci—along with infarct volume and spatial distribution parameters. This strategy transforms nuanced imaging findings into high-dimensional features recognizable by machine learning algorithms. The XGBoost model demonstrated its strength in capturing complex nonlinear interactions among these features, thereby achieving high specificity in identifying infarcts within specific vascular territories. The ultimate output of this model is not intended to replace radiologists’ diagnoses but to serve as an efficient decision-support tool. In clinical practice, particularly in emergency departments or primary stroke centers, the model can provide rapid, automated analysis of NCCT scans, offering radiologists an objective, quantitative probability prediction of the involved infarct territory. This assists in faster and more confident localization of the culprit vessel, especially when early signs are atypical or diagnostic experience is limited. It has the potential to shorten the time delay in the “imaging recognition-to-clinical decision” pathway, thereby preserving valuable time for subsequent endovascular therapy or thrombolysis and optimizing the acute stroke management workflow.

Despite methodological innovations, this study has several limitations. First, the reliance on single-center data with a limited sample size—only 118 patients were enrolled from one center, and some subgroups (e.g., the PCA group with merely 28 patients) had even smaller sample sizes—may restrict the model’s generalizability. This highlights the need for future multi-center collaborations to expand the sample size, which is further supported by post-hoc power analysis confirming insufficient statistical power in the PCA subgroup. Combined with uneven class distribution, highly distinguishable quantitative NCCT imaging features, and the inherent limitation that SMOTE-synthesized samples cannot fully mimic real clinical images, these factors collectively contributed to a perfect training-set AUC of 1.0 and mild overfitting in our models. While the adopted nested repeated stratified cross-validation (10 repetitions) effectively alleviated excessive overfitting, formal feature pruning and corresponding sensitivity analysis will be conducted in follow-up multi-center research after sample expansion. To address this issue, we have established collaborations with two tertiary hospitals and plan to prospectively enroll an additional ~330 patients (including multi-territory and basilar infarctions) from July 2025 to June 2028 for subsequent model reconstruction and validation. Second, the study only included PCA infarcts within the posterior circulation, excluding those involving the basilar artery territory; this may limit the model’s generalizability to complete posterior circulation stroke. Third, the model was only validated on an internal dataset—external validation in an independent cohort is essential to confirm its clinical applicability and robustness. Additionally, techniques such as transfer learning could help mitigate sample size limitations. Furthermore, developing real-time interactive diagnostic tools and integrating the model into clinical workflows may accelerate decision-making efficiency and provide better treatment opportunities for stroke patients.

## 5 Conclusion

This study developed and validated NCCT quantitative imaging features-based machine learning models for rapid AIS infarct territory localization. Using a “region-specific stratified modeling framework” strategy with TotalSegmentator for precise vascular segmentation, XGBoost outperformed LR in distinguishing ACA, MCA, and PCA infarctions, delivering superior specificity and AUCs, while LR offered greater stability and interpretability. XGBoost effectively captured subtle, imperceptible nonlinear NCCT features, rendering NCCT-based automated localization a cost-effective, accessible auxiliary tool to optimize triage within the critical therapeutic window-especially in primary care settings without advanced multimodal imaging. Future research should focus on external validation and integrating dynamic clinical biomarkers to enhance model robustness and applicability.

## Data Availability

The raw data supporting the conclusions of this article will be made available by the authors, without undue reservation.
